# Detection of foot at risk of ulceration using a new version of the sisped software in type 2 diabetes patients in a primary care setting

**DOI:** 10.1186/1758-5996-7-S1-A256

**Published:** 2015-11-11

**Authors:** Karla Freire Rezende, Naira Horta Melo, Thaisa de Fátima Almeida Rocha, Leila Maciel de Almeida e Silva, Helenalda Ferreira

**Affiliations:** 1 Universidade Federal de Sergipe, Brazil

## Background

The diabetic foot continues to generate high financial and psychological costs and a lot of preventive actions have been tried in an attempt to achieve better indicators. In Sergipe, the software SISPEDR was created to facilitate the foot examination of the patient with diabetes mellitus, and due to the need of some update, a new version was made.

## Objective

This study aimed to stratify the foot at risk in the first patients who used the second version of SISPED.

## Materials and methods

We evaluated 1076 patients assisted in the primary care set (SUS). The research was conducted by personal data collection and clinical protocol, evaluating signs, symptoms and test Results following the latest recommendations of the Brazilian Diabetes Society (SBD) and the International Consensus of Diabetic Foot.

## Results

Patients had an average age of 63,10±12,32 yrs., predominantly female (63.5%), and all of them had type 2 diabetes. The mean of HbA1C was 8,31%±2.03. Most of them had a foot at risk 1 (60,9%) and only 9,8% had risk 0. Risk 2 was present in 39% of the patients and 8,7% of the patients had risk 3. The presence of active ulcers was 3,2% and scaly foot, mycosis and ringworm were present in approximately 70% of patients.

## Discussion

Our study has shown a significant amount of diabetic patients with foot at risk in accordance with the reality of developing countries, which reinforces the need for intensive prevention of the diabetic foot. The SISPED, in its second version, was shown to be effective in helping to prevent the diabetic foot and to choose the most appropriate care.

## Conclusion

Given the current reality, intensive interventions are mandatory to contain the progression of the damage resulting from diabetic foot complications. It is in this context that the SISPED, now in its second edition, has proved to be an effective tool in minimizing the impact of diabetic foot.

